# The Role of Neprilysin and Insulin-Degrading Enzyme in the Etiology of Sporadic Alzheimer's Disease

**DOI:** 10.1523/JNEUROSCI.2152-24.2025

**Published:** 2025-04-29

**Authors:** Takahiro Morito, Shoko Hashimoto, Risa Takamura, Naoto Watamura, Naomasa Kakiya, Ryo Fujioka, Naomi Mihara, Misaki Sekiguchi, Kaori Watanabe-Iwata, Naoko Kamano, Mohan Qi, Yukio Matsuba, Satoshi Tsubuki, Takashi Saito, Nobuhisa Iwata, Hiroki Sasaguri, Takaomi C. Saido

**Affiliations:** ^1^Laboratory for Proteolytic Neuroscience, RIKEN Center for Brain Science, Saitama 351-0198, Japan; ^2^Pioneering Research Division, Medical Innovation Research Center, Shiga University of Medical Science, Otsu 520-2192, Japan; ^3^Dementia Pathophysiology Collaboration Unit, RIKEN Center for Brain Science, Saitama 351-0198, Japan; ^4^Department of Genome-based Drug Discovery & Unit for Brain Research, Graduate School of Biomedical Sciences, Nagasaki University, Nagasaki 852-8521, Japan; ^5^Department of Neurocognitive Science, Institute of Brain Science, Nagoya City University Graduate School of Medical Sciences, Nagoya 467-8601, Japan; ^6^Department of Neuroscience and Pathobiology, Research Institute of Environmental Medicine, Nagoya University, Nagoya 464-8601, Japan

**Keywords:** Abeta, Alzheimer, degradation, GWAS, mutation, neprilysin

## Abstract

An age-dependent decline in the amyloid-β (Aβ)-degrading enzyme neprilysin (NEP) has been implicated in the pathogenesis of sporadic Alzheimer's disease (AD). Recently identified risk alleles in the NEP-coding gene further support its role in AD etiology. However, evidence for the impact of NEP on the pathophysiological progression of Aβ plaque formation, particularly in comparison with another Aβ-degrading enzyme, insulin-degrading enzyme (IDE), is still lacking. Furthermore, the functional impact of the NEP mutation, M8V, caused by the AD risk allele in the NEP gene, remains unexplored. Here we found that NEP deficiency in *App^NL-F^* mice accelerates Aβ plaque formation more prominently than IDE deficiency in both male and female mice. Additionally, NEP/IDE double knock-out further exacerbated the plaque deposition of *App^NL-F^* mice, demonstrating a synergistic effect between the two enzymes. We also revealed that the M8V mutation in NEP reduced extracellular Aβ degradation in SH-SY5Y neuroblastoma cells, not by impairing catalytic activity but by increasing phosphorylation at an intracellular serine residue. This alteration in phosphorylation decreases NEP localization on the cell surface and extracellular vesicles, thereby limiting extracellular Aβ degradation. These observations point to the role of aging-associated neprilysin decline in sporadic AD pathogenesis and endorse the strategy of upregulating neprilysin activity to treat preclinical AD.

## Significance Statement

Neprilysin (NEP) is a key amyloid-β (Aβ)-degrading enzyme in the brain, but its role in the pathophysiological progression of Aβ plaque formation remains controversial, particularly in comparison with another Aβ-degrading enzyme, insulin-degrading enzyme (IDE). Here, we show that NEP deficiency in *App^NL-F^* mice accelerates Aβ plaque formation more prominently than IDE deficiency. This effect is further exacerbated in NEP/IDE double knock-out mice, demonstrating a synergistic relationship between the two enzymes. Moreover, the AD-associated NEP M8V mutation reduces extracellular Aβ degradation in neuroblastoma cells. These observations point to the role of aging-associated neprilysin decline in sporadic AD pathogenesis and endorse the strategy of upregulating neprilysin activity to treat preclinical AD.

## Introduction

Alzheimer's disease (AD) is the major cause of dementia that deprives patients of their quality of life and human dignity as the disease progresses. A large body of pathological and genetic evidence has established that the deposition of amyloid β peptide (Aβ) in the brain serves as a primary cause of this disorder, preceding the onset of clinical AD by more than two decades (Bateman et al., 2012; Selkoe and Hardy, 2016). AD is classically divided into two types: familial AD (FAD), which develops from a strong genetic background, and sporadic AD (SAD), which arises from both heritable and acquired factors such as aging and lifestyle (Gatz et al., 2006). While FAD cases caused by mutations in the amyloid precursor protein (APP) and presenilin 1/2 genes are linked to increased anabolism of Aβ (particularly Aβ42 and Aβ43), the mechanisms underlying Aβ accumulation in the etiology of SAD remain elusive. Because proteostasis is principally governed by the balance between anabolism and catabolism, and given that the anabolism of Aβ appears to be unaffected prior to the Aβ deposition that leads to SAD development (Saido, 2024), a plausible candidate cause of SAD is a decrease in Aβ catabolism (Saito et al., 2003; Saido, 2003b; Mawuenyega et al., 2010; Patterson et al., 2015). This notion led us to identify neprilysin (NEP) as a major Aβ-degrading enzyme in vivo (Iwata et al., 2000, 2001), while other groups identified insulin-degrading enzyme (IDE) encoded by the *IDE* gene as a contributing factor (Qiu et al., 1998; Farris et al., 2003; Leissring et al., 2003).

NEP is a type II membrane protein that exposes its C-terminal catalytic domain to the extracellular space (Turner et al., 2000), and its localization in cells is reportedly affected by phosphorylation of the N-terminal intracellular domain (Kakiya et al., 2012). Given that a deficiency of the *Mme* gene encoding NEP in the mouse brain resulted in a twofold increase in Aβ40 and Aβ42 (Iwata et al., 2001), and considering the number of studies consistently demonstrating an aging-dependent or AD-associated decline of NEP expression in the human and mouse brain (Iwata et al., 2002; Wang et al., 2003; Russo et al., 2005; Hellstrom-Lindahl et al., 2008), the aging-associated decrease in brain NEP expression likely plays a role in the pathogenesis of SAD (Iwata et al., 2001, 2005). Notably, the group led by Nordberg showed that NEP mRNA levels start to decrease approximately after the age of 50 in the normal human temporal and frontal cortices (Hellstrom-Lindahl et al., 2008). Figure S1 demonstrates an aging-dependent decline of neprilysin expression both in the human temporal cortex and mouse hippocampus. However, the relative importance of NEP and IDE in the development of AD remains controversial, and a side-by-side comparison of their effects in vivo is needed to assess each enzyme's impact on physiological and pathological Aβ catabolism. Incidentally, there are some other Aβ-degrading enzymes identified such as angiotensin-converting enzyme, endothelin-converting enzymes 1/2, cathepsin D, matrix metalloproteases 2/9, coagulation factor XI, and plasmin (Saido, 2003a; Saido and Leissring, 2012) although their contribution to Aβ metabolism appears relatively small.

A recent genome-wide association study (GWAS) identified two variants of the *MME* gene as risk alleles significantly associated with SAD (Bellenguez et al., 2022). This is remarkable because the association between NEP and AD pathogenesis had never been evidenced by human genetic studies before. These mutations had likely been overlooked presumably because the number of mutation carriers is relatively small (Bellenguez et al., 2022). The *MME* gene possesses 24 exons spanning over 80 kb, and the main transcript in the central nervous system (CNS) encodes a protein composed of 750 amino acid residues (Bayes-Genis et al., 2016). One risk variant is an intronic mutation and the other is a missense mutation that substitutes methionine at position 8 by valine (M8V). To our knowledge, the functional effects of the M8V mutation have not been investigated.

In the present study, we first investigated the relative contribution of NEP to in vivo Aβ catabolism in comparison with IDE. We crossbred *Mme* knock-out (KO) mice and *Ide* KO mice with an *App* knock-in mouse line, *App^NL-F^*, generating *App^NL-F^* × *Mme* KO, *App^NL-F^* × *Ide* KO, and *App^NL-F^* × *Mme/Ide* KO homozygous mouse lines to investigate their effects on Aβ pathology. The *App^NL-F^* mice harbor the Swedish (KM670/671NL) and Beyreuther/Iberian (I716F) mutations in the humanized mouse *App* gene and recapitulate typical Aβ pathology and neuroinflammation in brain tissue from ∼8 months of age (Saito et al., 2014). Our findings in mice indicate that NEP plays a greater role than IDE in in vivo Aβ catabolism amyloid plaques development. There was, however, an unexpected observation that IDE deficiency significantly aggravated Aβ pathology induced by NEP deficiency, indicating that NEP and IDE may synergistically be involved in Aβ metabolism. We subsequently examined the effect of the M8V mutation on the Aβ-degrading activity of NEP expressed in neuroblastoma cells and found that this mutation reduces NEP activity to degrade extracellular Aβ. This is likely due to the accelerated phosphorylation of serine at position 6 (S6) by the M8V mutation, which resulted in a decrease in the cell surface/extracellular localization of NEP. These observations suggest the role of an aging-associated decrease in brain NEP activity/expression in the pathogenesis of AD.

## Materials and Methods

### Animals

*Mme* KO mice (Lu et al., 1995) were kindly provided by Craig Gerard, Harvard Medical School, USA. *Ide* KO mice (Farris et al., 2003) were purchased from the Mutant Mouse Resource & Research Centers. *App^NL-F^* mice were described previously (Saito et al., 2014). *Mme* and *Ide* KO mice were crossbred with *App^NL-F^* mice to generate *App^NL-F^* × *Mme* KO and *App^NL-F^* × *Ide* KO mice, respectively. All mice used in this study were homozygous for the *App* mutations, NEP, and/or IDE deficiency on a C57BL/6J background. All mice were bred and maintained in accordance with regulations for animal experiments, including institutional guidelines and the ARRIVE guidelines, promulgated by the RIKEN Center for Brain Science [approval number: W2023-2-027(2)].

### Genotyping

Mouse tail tissue was placed in lysis buffer (10 mM Tris-HCl, pH 8.5; 5 mM EDTA, pH 8.0; 0.2% SDS; 200 mM NaCl; 20 µg/ml proteinase K) and incubated overnight at 55°C. After centrifugation at 4°C and 15,000 × *g* for 30 min, the supernatant was used for ethanol precipitation to collect genome DNAs. Genome DNAs were dissolved in distilled water and subjected to PCR. The primers used are described in Table S2.

### Brain sample preparation

Mice were anesthetized with isoflurane and transcardially perfused with ice-cold phosphate-buffered saline (PBS; Saito et al., 2014). Brains were extracted, maintained on ice, and dissected into two halves at the midline. For biochemical analyses, one hemisphere was divided into several parts including the cortex and hippocampus and stored at −80°C. For immunohistochemical analyses, brains were fixed with 4% paraformaldehyde in PBS, incubated at 4°C for 24 h, and rinsed with PBS until paraffin processing.

### Quantitative PCR for neuropeptide expression analysis

Total RNAs of WT mice (*n* = 6) and *Mme* KO (*n* = 6) were extracted from frozen cortical tissue using RNAiso Plus (Takara Bio). The RNAs were reverse-transcribed using ReverTra Ace qPCR RT Master Mix with gDNA Remover (Toyobo, FSQ-201) and applied to quantitative PCR using a QuantStudio 12K Flex Real-Time PCR system (Thermo Fisher Scientific), TB Green *Premix Ex Taq* II (Takarabio, RR820S), and a MicroAmp Optical 96-Well Reaction Plate (Applied Biosystems, N8010560). The forward (Fw) and reverse (Rv) primers used were as follows. Somatostatin Fw: CCCAACCAGACAGAGAATGATG, Rv: CCATTGCTGGGTTCGAGTT; neuropeptide Y Fw: TATCTCTGCTCGTGTGTTTGG, Rv: TCGCAGAGCGGAGTAGTAT; cholescystokinin Fw: GTCCGCAAAGCTCCTTCT, Rv: CATGTAGTCCCGGTCACTTATT, substance P Fw: CATGGCCAGATCTCTCACAAA, Rv: GCATCGCGCTTCTTTCATAAG. Primers were designed from RNA sequences of neuropeptide precursor protein transcripts.

### RNA sequencing

Total RNAs of wild type (WT) mice (*n* = 6) and *Mme* KO (*n* = 6) were extracted from frozen mouse cortex tissue using RNAiso Plus (Takara Bio) and the analysis was outsourced to Seibutsu Giken (Japan) for library construction, sequencing, and mapping to the reference genome (*Mus musculus*, GRCm39; https://www.ncbi.nlm.nih.gov/assembly/GCF_000001635.27). Identified mRNAs and their read counts were used to identify differentially expressed genes and to generate volcano plots using TCC-GUI (Su et al., 2019). For the gene ontology analysis, top-ranked upregulated or downregulated genes (within rank 1–2,000) were analyzed by ShinyGO (http://bioinformatics.sdstate.edu/go/; Ge et al., 2020) using the pathway database “GO biological process.”

### Behavioral analysis

In the open field test, each mouse was placed in the center of an open field maze (600 mm × 600 mm, O’Hara) and allowed to explore for 10 min. The time spent in the central region and the total distance traveled were measured as indicators of anxiety and locomotor activity, respectively. The Y-maze apparatus (O’Hara) was made of gray plastic and consisted of three arms (bottom width, 3 cm; top width, 10 cm; length, 40 cm; height, 12 cm) radiating from a central platform (3 cm × 3 cm × 3 cm triangle). Each mouse was placed at the center and allowed to explore freely for 8 min. A spontaneous alternation was counted when the mouse entered all three arms consecutively, serving as a measure of short-term working memory performance. Contextual fear conditioning was conducted over 4 consecutive days using an acrylic transparent square chamber (33 cm × 25 cm × 28 cm, O’Hara) with a metal grid floor (0.2 cm diameter, 0.5 cm spacing) and a transparent acrylic lid. Mice received an electric footshock (0.75 mA, 2 s) 4 min after placement in the chamber, and freezing behavior was recorded for a total of 5 min each day. Freezing was defined as movement of <20 pixels within a 0.5 s interval.

### Cell culture

SH-SY5Y cells obtained from the European Collection of Cell Cultures (94030304) were cultured in DMEM/F-12 (Invitrogen, 11320033), 10% (v/v) fetal bovine serum, and 1% penicillin/streptomycin (Merck, P4333) at 5% CO2, 37°C. Cells were counted using a Disposable Cell Counting Plate (Watson, 177-112C).

### Cell surface biotinylation/pull-down assay

To isolate cell surface proteins, cells were washed twice with ice-cold PBS containing 1 mM MgCl₂ and 0.1 mM CaCl₂ (PBS-CM) and then incubated with 0.5 mg/ml EZ-Link Sulfo-NHS-Biotin (Thermo Fisher Scientific, 21217) in PBS-CM at 4°C for 30 min. The biotinylation reaction was quenched by adding 50 mM NH₄Cl after removing the reaction solution, followed by incubation at 4°C for 10 min. Cells were then washed with PBS-CM and lysed in lysis buffer containing 100 mM Tris-HCl, pH 7.5, 1% Triton X-100, 0.15 M NaCl, PhosSTOP phosphatase inhibitor cocktail (Roche, 4906845001), and cOmplete protease inhibitor cocktail (EDTA-free; Roche, 11836170001). The lysates were incubated at 4°C for 1 h and then centrifuged at 15,000 × *g* for 30 min. The resulting supernatants were used for biotinylated protein isolation. Magnetic streptavidin-conjugated beads (Dynabeads M-280 Streptavidin, Thermo Fisher, DB11205) were added to the cell lysates at a ratio of 5 µg protein per mg beads, diluted in PBS containing 0.1% BSA (PBSB). The mixture was incubated at 4°C for 1 h. Beads were then washed five times with PBSB and used for Western blot analysis.

### Western blot

Mice brain tissues were homogenized in 50 mM Tris-HCl, pH 7.6, 0.15 M NaCl, and Complete protease inhibitor cocktail (Roche, 11697498001). We confirmed that NEP was active in EDTA-free protease cocktail, but inactive in protease cocktail containing EDTA, as described in the supplier's specification. Cells were homogenized in lysis buffer described above. Homogenates were incubated at 4°C for 1 h, centrifuged at 15,000 × *g* for 30 min, and the supernatants were collected (Saito et al., 2014). Equal amounts of protein per lane were subjected to SDS-PAGE and transferred to PVDF or nitrocellulose membranes (Invitrogen). To load bead-bound proteins, the samples were boiled in SDS-PAGE sample buffer at 95°C for 5 min. To detect APP-CTFs, delipidated samples were loaded and the membrane was boiled for 5 min in PBS before the next step. After washing (10 min in TBST) and blocking (1 h using Amersham ECL Blocking Agent, Cytiva) at room temperature, the membrane was incubated at 4°C overnight with primary antibodies. After washing the membrane three times with TBST for 10 min, secondary antibodies were applied and incubated for 1 h at room temperature. The target protein was subsequently visualized using ECL Select (Cytiva) and detected with a Luminescent Image Analyzer LAS-3000 Mini (Fujifilm). The primary antibodies utilized in this study were as follows: anti-APP (full-length; 1:1,000, Millipore, 07-667), anti-APP-CTFs (1:1,000, Sigma-Aldrich, A8717), anti-NEP (1:500, R&D Systems, AF1182; or 1:1,000, Leica, CD10-270-L-CE, 56C6), anti-NEP (pS6; a lab-made rabbit antibody raised against S6-phosphorylated NEP 1–28 peptides; Kakiya et al., 2012), anti-IDE (1:1,000, Abcam, ab32216), anti-GAPDH, HRP-conjugated (1:15,000, HRP-60004), and anti-β-actin, HRP-conjugated (1:15,000, HRP-66009). Secondary antibodies employed were as follows: Anti-Mouse IgG, HRP-Linked F(ab’)₂ Fragment (1:5,000, NA9310, Cytiva) and Anti-Rabbit IgG, HRP-Linked F(ab’)₂ Fragment (1:5,000, NA9340, Cytiva).

### Quantification of Aβ40 and Aβ42 in the mouse brain

**Figure 1.**
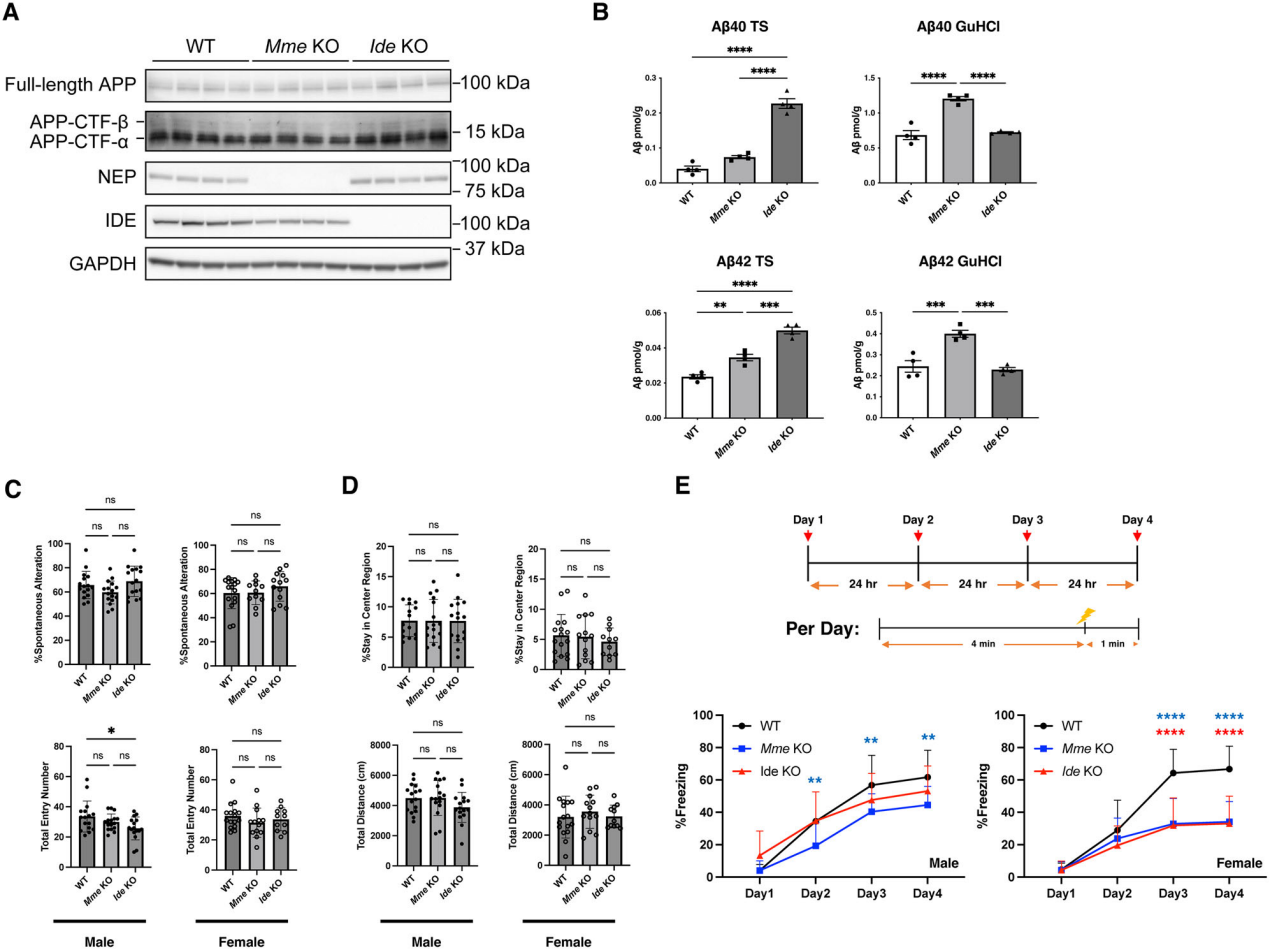
Cortical Aβ levels and mouse behavioral analysis of NEP- or IDE-deficient mice. ***A***, Normal APP processing and deficiency of NEP or IDE in *Mme* or *Ide* KO mice confirmed by Western blot (*n* = 4). ***B***, ELISA quantification of Aβ40 and Aβ42 in Tris-buffered saline (TS)-soluble and guanidine hydrochloride (GuHCl)-soluble fractions of brain homogenates from 12-month-old wild-type (WT), *Mme* KO, and *Ide* KO mice (*n* = 4). ***C–E***, Results of battery of behavioral experiments (male: *n* = 16, female: *n* = 16 [WT], 13 [*Mme* KO], 12 [*Ide* KO]). ***C***, Y-maze behavioral analysis of WT, *Mme* KO, and *Ide* KO mice. ***D***, Open field behavioral analysis of WT, *Mme* KO, and *Ide* KO mice. ***E***, Contextual fear conditioning test of WT, *Mme* KO, and *Ide* KO mice. Error bars: standard errors of the mean, *p < 0.05, **p < 0.01, ***p < 0.001, ****p < 0.0001 when comparing three groups by one-way ANOVA.

**Figure 2.**
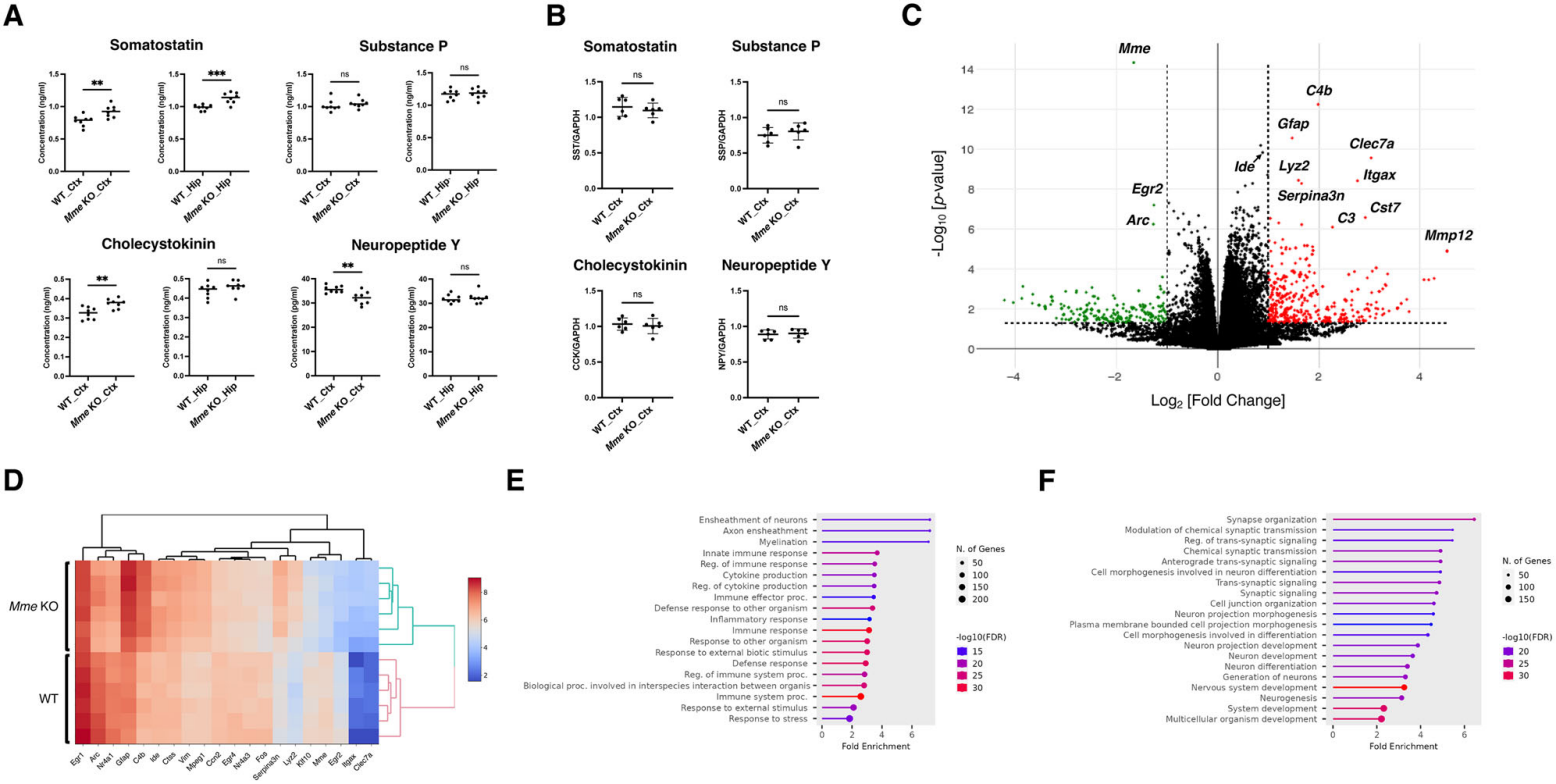
Alterations of neuropeptide levels and transcriptomes in *Mme* KO mice. ***A***, ELISA quantification of four neuropeptides in brain homogenates from cortex (Ctx) or hippocampus (Hip) of WT and *Mme* KO mice (*n* = 4/4 [male/female]). ***B***, Quantitative PCR analysis of each neuropeptide transcript (*n* = 3/3 [male/female]). ***C***, Comparison of expressed genes in WT and *Mme* KO mouse brain cortex (*n* = 6, male) identified by RNA sequencing. ***D***, An expression heat map of 10 representative upregulated and downregulated genes. ***E***, Gene ontology analysis of upregulated genes. ***F***, Gene ontology analysis of downregulated genes. Error bars: standard errors of the mean, ns: not significant, **p < 0.01, ***p < 0.001 when comparing groups by *t* test.

An Optima MAX-XP Ultracentrifuge (Beckman Coulter) with a TLA 110 rotor (Beckman Coulter, 366735) was used for ultracentrifugation. Brain tissues were homogenized in 5× brain volume of buffer A (50 mM Tris-HCl, pH 7.6, 150 mM NaCl and cOmplete protease inhibitor cocktail) at 50 mg/ml using a medical beads shocker (Saito et al., 2014). The homogenized samples were directed to centrifugation at 200,000 × *g* for 20 min at 4°C, and the supernatant was collected as a TS-soluble fraction. The pellet was loosened with buffer A, centrifuged at 200,000 × *g* for 5 min at 4°C, and then dissolved in 1× brain volume of 6 M GuHCl buffer after removal of the supernatant. After incubation at room temperature for 1 h, the sample was sonicated at 25°C for 1 min. Subsequently, the sample was centrifuged at 200,000 × *g* for 20 min at 25°C and the supernatant was collected and diluted with 11× Buffer A (GuHCl fraction). TS and GuHCl fractions (100 µl each) were loaded onto 96-well plates and incubated at 4°C overnight using Human βAmyloid (1–40) ELISA Kit Wako (FujiFilm Wako, 292-62301) and Human βAmyloid (1–42) ELISA Kit Wako (FujiFilm Wako, 298-62401) according to the manufacturer's instructions. These ELISA kits detect both murine and human Aβ species as shown in Figures 1*B* and 2*C*.

### Quantification of Aβ40 and Aβ42 in cell culture medium

Synthetic Aβ40 and Aβ42 were purchased from Peptide Institute (4307-v, 4349-v), dissolved in DMSO to 2 mg/ml, and used for subsequent experiments with appropriate dilution. SH-SY5Y cells were seeded in 12-well plates at a density of 3.0 × 10^5^ cells/well and incubated for 1 d at 37°C in 5% CO_2_. The cells were then transfected with pcDNA 3.1 (+) mammalian expression vector (Invitrogen, V79020), encoding human NEP with or without mutations S6A, M8V, and S6A/M8V, using FuGENE 4K Transfection Reagent (Promega, E5911), according to the manufacturer's instructions. After 5 h, 50 nM Aβ40 or Aβ42, prepared in medium, was added to each well to a final concentration of 500 pM. The cells were incubated for 3 d to allow NEP to degrade the Aβ species. Afterward, the cultured medium was collected and centrifuged at 15,000 × *g* for 1 min. To prevent Aβ fibrillization and to dissociate the amyloid aggregates, the resulting supernatant was treated with guanidine hydrochloride to a final concentration of 500 mM. This solution was subsequently used for the ELISA assay as described above.

### Immunostaining

Paraffin-embedded mouse brain sections were subjected to deparaffinization and then antigen retrieval was performed by autoclave processing at 121°C for 5 min (Saito et al., 2014). After inactivation of endogenous peroxidases in a 0.3% H_2_O_2_ solution for 30 min, the sections were washed with TNT buffer (0.1 M Tris, pH 7.5, 0.15 M NaCl, 0.05% Tween20), blocked for 30 min in TNB buffer (0.1 M Tris, pH 7.5, 0.15 M NaCl) with 0.5% blocking reagent (AKOYA Biosciences, FP1012), and incubated overnight at 4°C in the same buffer with primary antibodies. The primary antibodies used were as follows: Aβ1–5 (N1D; 1:200, lab-made; Saido et al., 1994), Aβ (82E1; 1:200, Immuno-Biological Laboratories, #10323), GFAP (1:200, Millipore, AB5804), Iba1 (1:200, Fujifilm Wako, 013-27691), synaptophysin (1:200, PROGEN, 61012), PSD95 (1:50, Synaptic Systems, 124-011), Lamp1 (1:200, Abcam, ab24170), and BACE1 (1:200, Cell Signaling, 5606S). After washing the sections three times with TNT for 5 min, secondary antibodies [Goat anti-Mouse IgG (H + L) Alexa Fluor 488, 1:200; Goat anti-Rabbit IgG (H + L) Alexa Fluor 555, 1:200] were applied. Before mounting, the sections were treated, when necessary, with Hoechst33342 (Invitrogen, H3570) diluted to 1:5,000 in PBS. Data images were obtained using a NanoZoomer Digital Pathology C9600 (Hamamatsu Photonics) or VS200 (Evident). Immunoreactive signals were quantified by Definiens Tissue Studio (Definiens) or MetaMorph (Molecular Devices).

### Neprilysin activity measurement

One microgram of protein from transfected cell lysates was incubated in an assay mixture containing cOmplete Protease Inhibitor Cocktail (Roche, 5056489001), 10 µM Z-Leu-Leu-Leu-H (aldehyde; Peptide Institute, 3175-v), and Suc-Ala-Ala-Pro-Phe-MCA (Peptide Institute, 3114-v) in 50 mM MES buffer, pH 6.5, for 1 h at 37°C, followed by incubation on ice for 5 min. To serve as a negative control, a neprilysin inhibitor, thiorphan (Cayman Chemical, 76721-89-6), was added to the mixture at a concentration of 10 µM. The reaction was quenched by adding phosphoramidon (Peptide Institute, 4082) and leucine aminopeptidase (Merck, L5006) at concentrations of 20 µM and 10 µg/ml, respectively, and incubation performed for an additional hour at 37°C followed by incubation on ice for 5 min. Enzymatic activity was halted by adding EDTA to a final concentration of 10 mM, and fluorescence was measured using a plate reader (infinite M1000 PRO, TECAN) with excitation at 390 nm and emission at 460 nm. AMC (7-Amino-4-methylcoumarin, Peptide Institute, 3099-v) was utilized as a standard.

### Immunoisolation of EVs using magnetic beads

Magnetic beads were prepared using the Dynabeads Antibody Coupling Kit (DB14311, Thermo Fisher) and coated with an anti-CD9 antibody (ab236630, Abcam) according to the manufacturer's protocol. The coated beads were incubated with 500 µl of culture medium at 4°C overnight, followed by three washes with PBS. The captured EVs were then analyzed by Western blot, NEP activity assay, and immunofluorescence. For the NEP activity assay, the beads were removed before fluorescence measurement. For immunofluorescence, the beads were incubated with primary antibodies—goat anti-NEP (1:200, AF1182, R&D Systems) and mouse anti-CD63 (1:200, ab8219, Abcam)—at room temperature for 1 h. After washing, they were incubated with secondary antibodies—donkey anti-mouse IgG Alexa Fluor 647 (1:200, AP192SA6, Sigma-Aldrich) and donkey anti-goat IgG (H + L) Alexa Fluor Plus 555 (1:200, A32816, Invitrogen). Following three PBS washes, the beads were resuspended in PBS, mounted on a small enclosed glass slide surrounded by tape, and covered with a coverslip for imaging using bench-top confocal fluorescence microscopy (BC43, Oxford Instruments).

### Statistics

All data are shown as the mean ± SEM. For comparisons between two groups, statistical analyses were conducted by Student's *t* test. For comparisons among three or more groups, one-way analysis of variance (ANOVA) was used followed by Tukey's multiple comparisons test. These analyses were performed using GraphPad Prism 10 software (GraphPad software). The levels of statistical significance are shown as *p* values: *p < 0.05, **p < 0.01, ***p < 0.001, and ****p < 0.0001.

## Results

### *Mme* and *Ide* KO mice exhibit distinct properties of endogenous Aβ degradation in the brain

*Mme* KO and *Ide* KO mouse lines lack expression of NEP and IDE, respectively, and exhibit normal APP processing profiles (Fig. 1*A*). APP is initially cleaved either by α-secretase or β-secretase, generating the C-terminal fragments (CTFs) CTF-α and CTF-β, respectively (O'Brien and Wong, 2011). There was no significant difference in the amounts of full-length APP or APP-CTFs in WT, *Mme*, or *Ide* KO mice, indicating that a deficiency of NEP or IDE does not affect the production or α/β-secretase-mediated processing of APP at the level seen in Western blot.

To evaluate the roles of NEP and IDE in the physiological degradation of Aβ in vivo, we compared murine Aβ profiles in the brains of single *Mme* KO and *Ide* KO by enzyme-linked immunosorbent assay (ELISA; Fig. 1*B*). The ELISA kits used detect both murine and human Aβ species and recognize Aβ using N-terminal or C-terminal specific antibodies as capture and detection antibodies, allowing distinction between Aβ40 and Aβ42. Whole-brain proteins were extracted by Tris-buffered saline (TS) followed by guanidine hydrochloride (GuHCl) for ELISA quantification of TS-soluble/GuHCl-soluble Aβ species (Sasaguri et al., 2022). It should be noted that ELISA of TS-soluble fractions may underestimate Aβ levels due to the concealed C-terminal structure of soluble Aβ aggregates. Additionally, the GuHCl-soluble fractions contain both insoluble and membrane-bound Aβ species. *Mme* KO mice showed increases in TS-soluble Aβ42, and GuHCl-soluble Aβ40 and Aβ42 (1.5-, 1.8- and 1.6-fold, respectively), in contrast with increases of TS-soluble Aβ40 and Aβ42 (5.6- and 2.1-fold) and unchanged GuHCl-soluble Aβ40 and Aβ42 in *Ide* KO mice. These results indicate that NEP mainly degrades GuHCl-soluble Aβ species in preference to TS-soluble Aβ species and that the main role of IDE in this context is to degrade TS-soluble Aβ species. It is possible that the IDE-sensitive TS-soluble Aβ40 and Aβ42 could be more toxic than the NEP-sensitive Aβ species (Walsh and Selkoe, 2004, 2016; Shankar et al., 2008). Of note, the quantity of GuHCl-soluble Aβ40 and Aβ42 was markedly greater than that of TS-soluble Aβ40 and Aβ42 in WT, *Mme* KO, and *Ide* KO mouse brain cortices. This indicates the increased amounts of total brain Aβ40 and Aβ42 in *Mme* KO mice compared with WT and *Ide* KO mice.

Then we performed behavioral tests (Y-maze, open field, contextual fear conditioning) to analyze cognitive phenotypes of the two KO mouse strains. We observed no difference in Y-maze and open field tests except for a slight decrease in the total entry number for male *Ide* KO mice (Fig. 1*C*,*D*). However, we found that both male and female *Mme* KO mice exhibited a lower freezing rate for fear conditioning than WT mice, indicating their long-term memory impairment (Fig. 1*E*). Additionally, female *Ide* KO mice also showed lower freezing rates.

### *Mme* KO mice exhibit subtle changes in neuropeptide concentrations but marked transcriptional alterations

To further investigate the mechanism of *Mme* KO memory impairment, we used brain extracts containing the cortex or hippocampus from WT and *Mme* KO mice to perform ELISA experiments on four neuropeptides (somatostatin, substance P, cholecystokinin and neuropeptide Y) that are considered to be substrates of NEP in the CNS at least in a test-tube paradigm (Turner et al., 2001; Saito et al., 2003; Fig. 2*A*). We observed slight increases in cortical and hippocampal somatostatin and cortical cholecystokinin levels and a slight decrease in cortical neuropeptide Y. The effect of NEP deficiency on the levels of these neuropeptides was negligibly smaller than on that of Aβ40 and Aβ42, ranging between −10 and 20% (Table S1; Iwata et al., 2001). We also measured the quantity of mRNA of the four peptides’ precursor proteins and confirmed no changes (Fig. 2*B*), indicating that the slight changes in the levels of these neuropeptides were caused by alterations to post-translational metabolism. Consistently, immunohistochemical staining for somatostatin, substance P, cholecystokinin, and neuropeptide Y showed minimal differences in the levels of these neuropeptides between WT and *Mme* KO mice (Fig. S2). This further supports the conclusion that NEP plays a relatively minor role in the catabolism of these neuropeptides, though it should be noted that immunohistochemical quantification is less sensitive than ELISA (see Discussion for more details).

We also performed comprehensive RNA sequencing using mRNA extracted from WT and *Mme* KO mouse cortical tissue and identified differentially expressed genes consisting of 624 upregulated and 340 downregulated genes (Fig. 2*C*,*D*). Gene ontology analysis revealed that immunoinflammatory genes (e.g., Gfap, C3, C4b) were upregulated, while neuronal development-involved genes (e.g., Egr2, Arc) were downregulated in *Mme* KO mice, possibly accounting for the anomalous long-term memory characteristic of the *Mme* KO mice (Fig. 2*E*,*F*).

### NEP deficiency accelerates Aβ amyloidosis in *App* knock-in mice, the effect of which is strengthened by additional IDE deficiency

**Figure 3.**
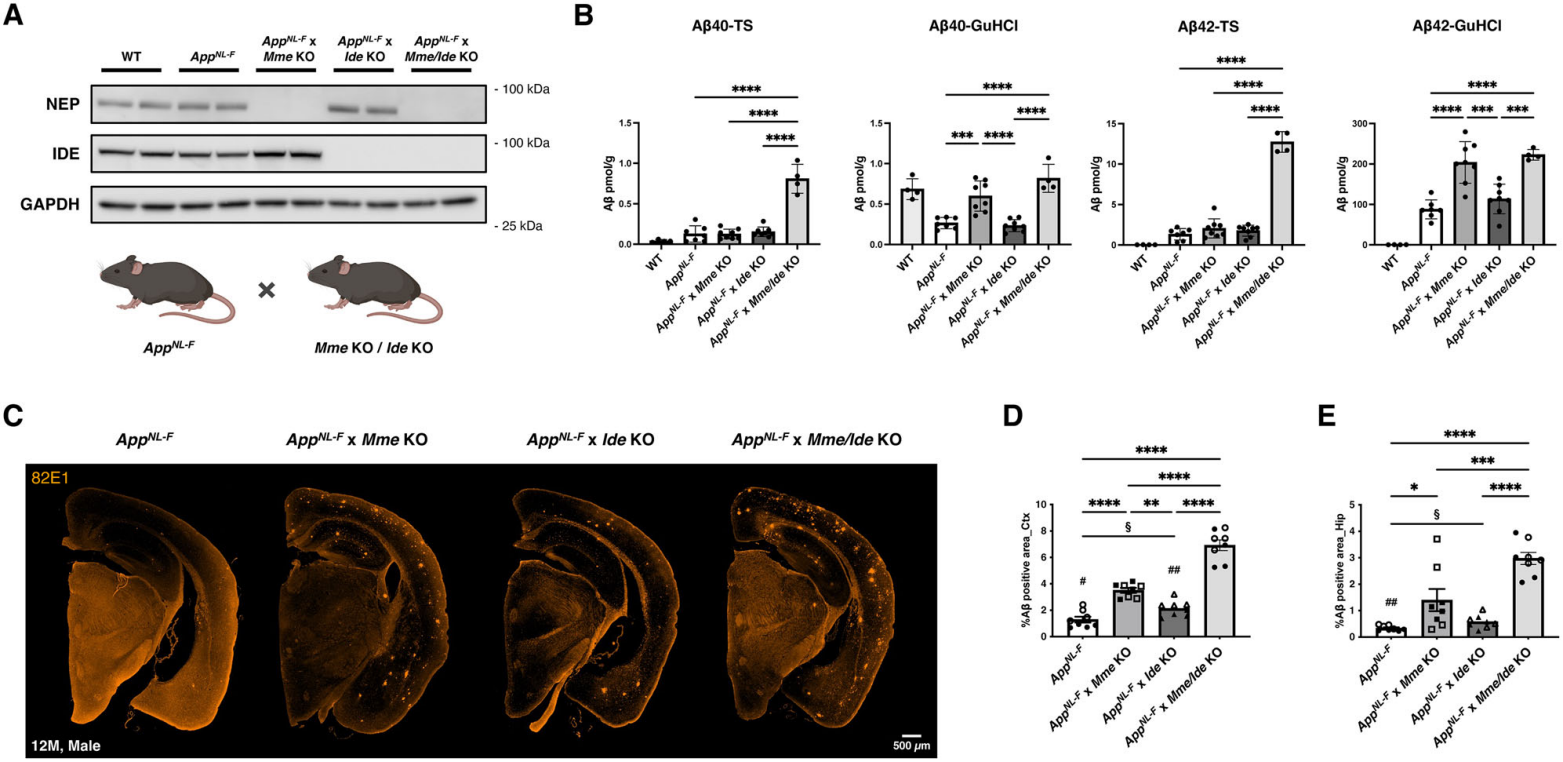
Effects of NEP and IDE deficiency on Aβ metabolism and pathology in 12-month-old *App^NL-F^* mice. ***A***, Confirmation of NEP and/or IDE deficiency in mice by Western blot. ***B***, ELISA quantification of Aβ40 and Aβ42 in brain cortical tissues extracted with Tris-buffered saline (TS) or guanidine hydrochloride (GuHCl). ***C***, Representative images of Aβ immunostaining (82E1 antibody) shown in orange (*n* = 8). Error bar: 500 µm. ***D***, ***E***, Quantification of Aβ immunopositive areas in the cortex (Ctx, ***D***) and hippocampus (Hip, ***E***). Closed and open symbols indicate male and female mice, respectively. ^#^*p* < 0.05; ^##^*p* < 0.01 when comparing male and female within the same group by unpaired Student's *t* test. ^§^*p* < 0.05 when comparing two groups, *App^NL-F^* and *App^NL-F^*/*Ide* KO by unpaired Student's *t* test. Error bars: standard errors of the mean, *p < 0.05, **p < 0.01, ***p < 0.001, ****p < 0.0001 when comparing four groups by one-way ANOVA.

To elucidate the roles of NEP and IDE in Aβ accumulation, we crossbred *Mme* and/or *Ide* KO mice with *App^NL-F^* mice, obtaining *App^NL-F^* × *Mme* KO, *App^NL-F^* × *Ide* KO, and *App^NL-F^* × *Mme*/*Ide* KO mouse lines. Western blot analyses confirmed NEP and/or IDE deficiency in these mice (Fig. 3*A*). We then quantified TS- and GuHCl-soluble Aβ40 and Aβ42 in brain tissue from 12-month-old mice (Fig. 3*B*). The *App*^NL-F^ mice start accumulating pathological Aβ in the brain between 9 and 12 months of age (Saito et al., 2014). In the TS-soluble fraction, the *App^NL-F^* × *Mme*/*Ide* triple mutant line exhibited significantly higher (over eight times) Aβ40 and Aβ42 levels than the other three lines, indicating that NEP and IDE play a synergistic role in Aβ metabolism, although the amount of Aβ42 in this fraction accounts for only 1–5% of total Aβ42. In the GuHCl-soluble fractions, *App^NL-F^* × *Mme* KO mice showed a more than twofold increased quantity of Aβ40 and Aβ42. Remarkably, there was no significant difference between *App^NL-F^* × *Mme* KO and *App^NL-F^* × *Mme*/*Ide* KO in this TS-soluble fraction, indicating that NEP plays a more important role in degrading GuHCl-soluble Aβ species. These findings indicate that NEP and IDE play analogous roles to degrade TS-soluble Aβ species and that only NEP plays a role in degrading GuHCl-soluble Aβ species. NEP being a membrane-bound peptidase is consistent with its preference for GuHCl-soluble to TS-soluble Aβ as a substrate. Consistently, a number of membrane-bound proteases prefer insoluble substrate proteins. They include α-, β- and γ-secretases. This is presumably because the enzyme-substrate interaction becomes more intimate when they exist in the vicinity of each other.

We next used immunohistochemical analyses to evaluate Aβ accumulation in 12-month-old animals from these four mouse lines. We found that *App^NL-F^* × *Mme* KO and *App^NL-F^* × *Mme/Ide* KO mice had significantly greater Aβ accumulation both in the cortex and hippocampus than that seen in *App^NL-F^* and *App^NL-F^* × *Ide* KO mice (Fig. 3*C–E*). A statistically significant difference was observed between *App^NL-F^* and *App^NL-F^* × *Ide* KO when compared by Student's *t* test, but not by one-way ANOVA, indicating that the effect of IDE deficiency was relatively low. However, additional *Ide* deficiency significantly augmented Aβ pathology in the NEP-deficient *App^NL-F^* mice. This suggests that an increase in TS-soluble Aβ species contributes to Aβ plaque formation. Female mice tended to show more Aβ deposits than male mice, with the difference being more significant in *App^NL-F^* (cortex and hippocampus) and *App^NL-F^* × *Ide* KO (cortex) mice.

### NEP and IDE deficiency drive pathological alterations mediated by Aβ plaques

**Figure 4.**
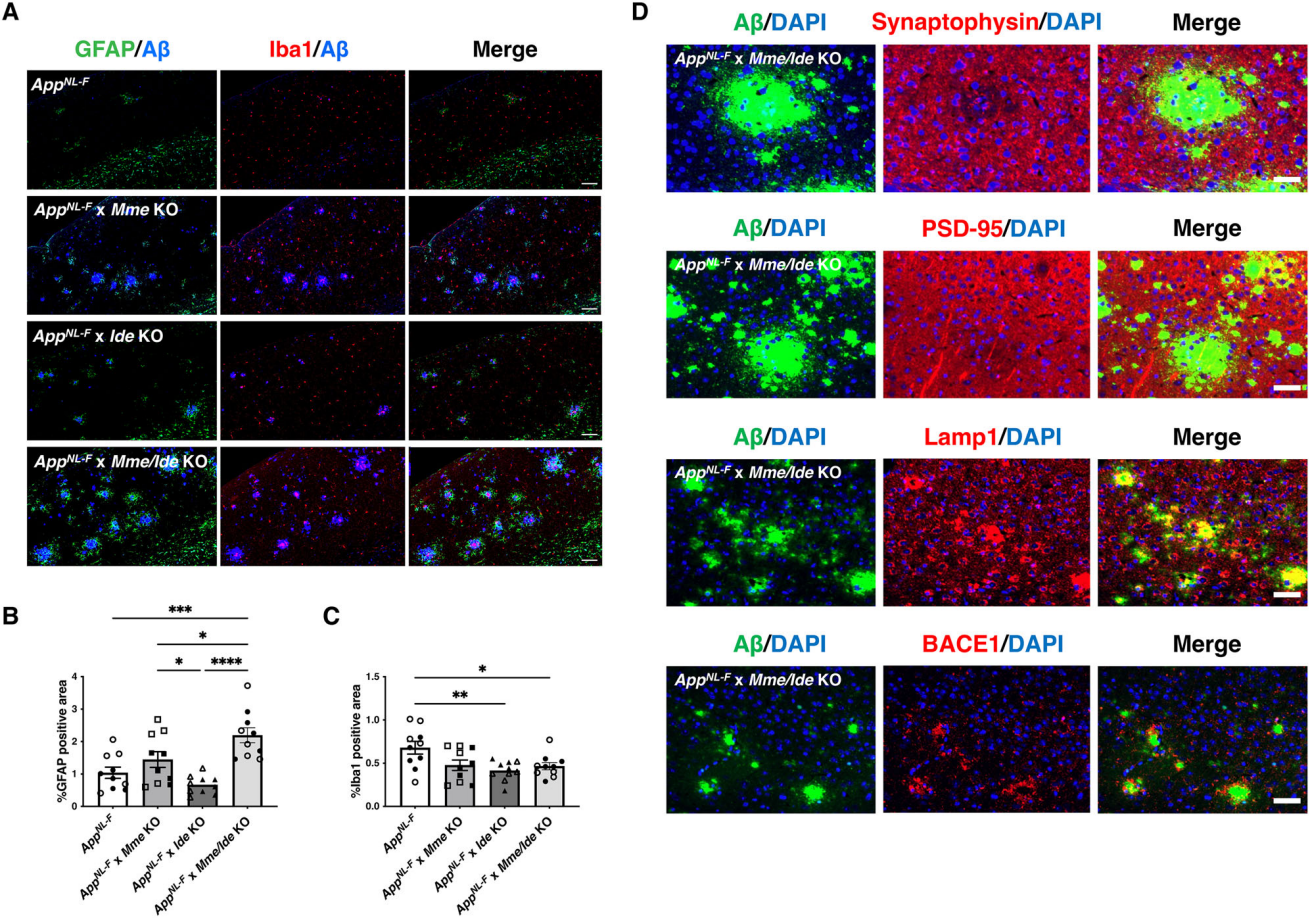
Neuroinflammation and pathological alterations induced by NEP and IDE deficiency-associated Aβ deposition. ***A–C***. Neuroinflammation in cortex of each mouse line represented by GFAP and Iba1 immunopositivity (***A***) and quantification of GFAP-positive (***B***) and Iba1-positive (***C***) areas. Blue: Aβ (82E1). Scale bars: 100 µm. ***D***, Representative immunostaining of PSD95, synaptophysin, Lamp1, and BACE1 in cortex of 12-month-old *App^NL-F^* × *Mme/Ide* KO mice. Aβ plaques were stained using N1D (PSD95 and synaptophysin) and 82E1 (Lamp1 and BACE1). Scale bars: 50 µm. Error bars: standard errors of the mean, *p < 0.05, **p < 0.01, ***p < 0.001, ****p < 0.0001 when comparing four groups by one-way ANOVA.

Neuroinflammation, characterized by reactive astrocytes and activated microglia surrounding Aβ plaques, is a prominent pathological feature observed in both AD patients and model mice (Saito et al., 2014; Bellenguez et al., 2020). To assess neuroinflammation, we performed immunohistochemistry using GFAP and Iba1 as markers of astrocytosis and microgliosis, respectively, across the four mouse lines (Fig. 4*A*). GFAP immunopositivity in the cortex was largely proportional to the presence of Aβ plaques (Fig. 4*B*), indicating astrocytosis associated with Aβ accumulation. The difference of GFAP-positive area between *App^NL-F^* and *App^NL-F^* × *Mme* KO mice was not statistically significant, although there was a trend toward an increase in *App^NL-F^* × *Mme* KO mice (*p* = 0.098). In contrast, Iba1 immunoreactivity in *App^NL-F^* mice tended to be higher compared with the other lines, despite a lower Aβ plaque burden (Fig. 4*C*). However, dense Iba1 signals, indicative of microgliosis, were consistently observed in the proximity of Aβ plaques in all models (Fig. 4*A*). These findings are consistent with a previous report that older *App^NL-F^* mice, which are expected to have more Aβ plaques, exhibit fewer Iba1-positive areas in the cortex than younger mice (Masuda et al., 2016). In addition, these neuroinflammatory phenotypes were also observed around Aβ plaques in the hippocampal region (Fig. S3). Moreover, we analyzed the localization of pre- and postsynaptic markers, synaptophysin and PSD95, in these models and found a loss of these markers in the vicinity of Aβ plaques, indicating synaptic alterations mediated by Aβ plaques, consistent with our previous study (Saito et al., 2014; Fig. 4*D*). These alterations became more severe with increased Aβ deposition, suggesting enhanced synaptic toxicity due to NEP deficiency (Fig. S4*A,B*). Additionally, we detected immunoreactivity for lysosomal-associated membrane protein 1 (Lamp1), a lysosomal marker, and beta-site APP cleaving enzyme 1 (BACE1) around plaques, indicating lysosomal abnormalities, impaired APP processing, and neuritic dystrophy triggered by Aβ plaques (Fig. 4*D*; Sadleir et al., 2016; Whyte et al., 2020). These observations were also present in the hippocampus and accelerated by NEP deficiency (Figs. S3, S4*C,D*).

Taken together, NEP contributes to the degradation of GuHCl-soluble, amyloidogenic Aβ in concert with IDE-mediated TS-soluble Aβ degradation, thereby reducing Aβ deposition in vivo. The role of NEP is crucial to suppressing the formation of Aβ plaques and concomitant pathological alterations such as gliosis, lysosomal abnormalities, impaired APP processing, and neuritic dystrophy.

### The M8V mutation decreases the Aβ-degrading activity of NEP expressed in SH-SY5Y cells

**Figure 5.**
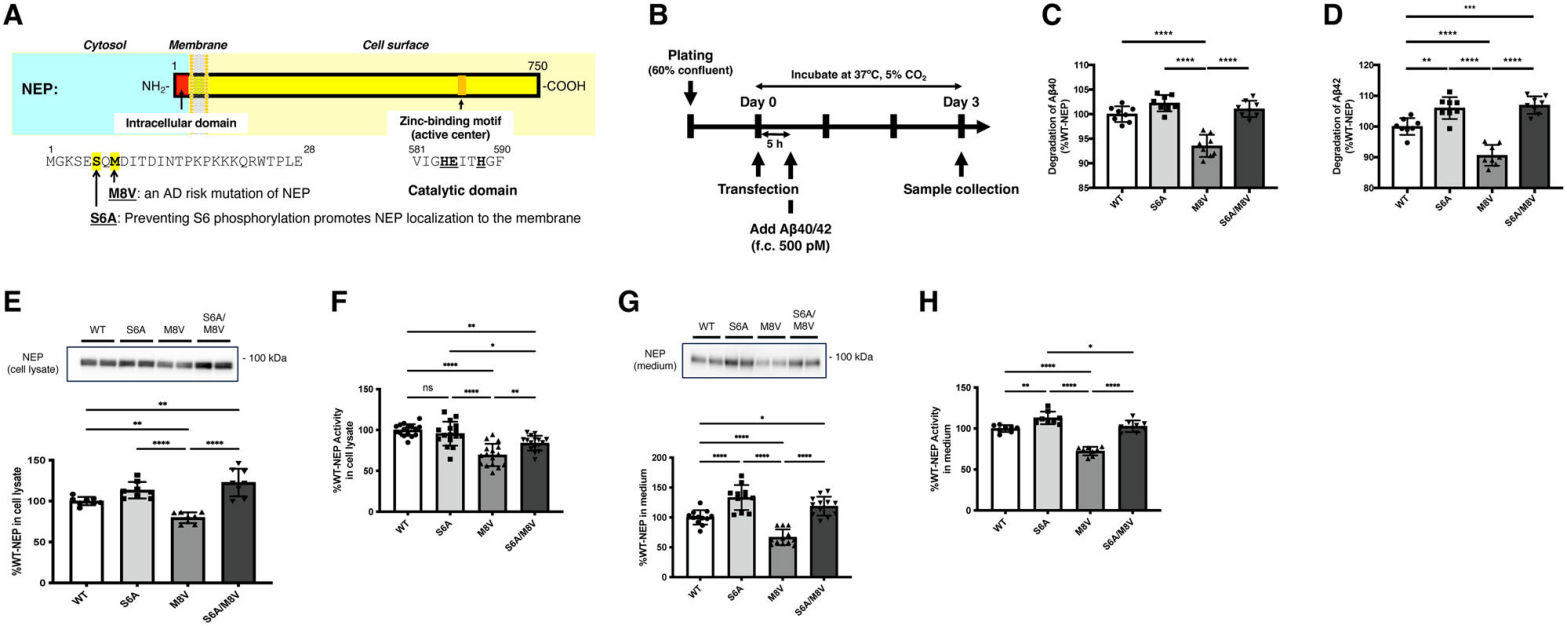
The AD risk-associated NEP mutation M8V impairs Aβ degradation activity. ***A***, Scheme for membrane-bound NEP structure and locations of variants. ***B***, Experimental timeline for NEP variant expression and Aβ degradation measurement in SH-SY5Y cells. f.c., final concentration. ***C***, ***D***, ELISA quantification of Aβ40 (***C***) or Aβ42 (***D***) in cell culture medium after 3 d of incubation (*n* = 8). ***E***, Western blot of NEP in cell lysate. ***F***, Standardized NEP activity in 1 µg of protein cell lysate. ***G***, Western blot of NEP in medium. ***H***, Standardized NEP activity in 10 µl medium. Error bars: standard errors of the mean, *p < 0.05, **p < 0.01, ***p < 0.001, ****p < 0.0001 when comparing four groups by one-way ANOVA.

The association of NEP with AD etiology has been supported by human genetic studies. Recently, a GWAS identified single-nucleotide variants within the *MME* gene, which encodes NEP (Bellenguez et al., 2022). One such risk allele, rs61762319 (chr3:155084189, A > G), exhibited a significant association with SAD (*p* = 2.2 × 10^−11^). This missense mutation replaces the eighth methionine of NEP with valine (M8V); however, how this mutation affects NEP function remains unexplored, despite its location being distant from NEP's catalytic domain (Fig. 5*A*). Our previous research demonstrated that the sixth serine of NEP (S6) phosphorylation modulates cellular localization, with the S6A mutation redirecting NEP from intracellular vesicles to the cell surface (Kakiya et al., 2012). Given that the M8V mutation is just two residues away from S6, we hypothesized that it might impair Aβ degradation by influencing S6 phosphorylation and suppressing NEP translocation to the cell surface.

To test this hypothesis, we expressed WT human NEP and its variants (S6A, M8V, and S6A/M8V) in SH-SY5Y cells via plasmid lipofection. The cell lysates were prepared using detergent-based lysis, ensuring the inclusion of both cytosolic and membrane proteins. Since nontransfected SH-SY5Y cells exhibit minimal NEP expression, we confirmed the successful expression of each NEP variant in cell lysates of transfected cells (Fig. S5*A*). Extracellular Aβ degradation was assessed by ELISA 3 d post-transfection (Fig. 5*B*). While the S6A mutation, which blocks S6 phosphorylation, enhanced Aβ degradation and reduced the Aβ42/Aβ40 ratio, the AD risk-associated M8V mutation significantly impaired extracellular degradation of both Aβ40 and Aβ42 without altering their ratio (Fig. 5*C*,*D*; Fig. S5*B–D*).

Western blot analysis showed that NEP levels in cell lysates were reduced for the M8V mutant but increased for the S6A and S6A/M8V mutants (Fig. 5*E*). NEP enzymatic activity was also reduced in the M8V cell lysates (Fig. 5*F*). Intriguingly, we detected NEP protein in the cell culture medium, where the M8V mutation led to a marked reduction in both NEP quantity and activity after 3 d of incubation (Fig. 5*G*,*H*). This reduction was more pronounced than the intracellular decrease, suggesting that the M8V mutation mainly suppresses NEP's extracellular localization (Fig. 5*E*,*F*). Moreover, introducing an additional S6A mutation to NEP M8V mitigated or reversed the M8V-induced effects, suggesting that S6 phosphorylation plays a role in M8V-mediated NEP impairment. However, Western blot analysis using a phospho-S6 (pS6)-specific antibody did not detect pS6 in cell lysates (Fig. S5*E*). Additionally, a cell surface biotinylation/pull-down assay revealed no significant changes in NEP levels on the cell surface (Fig. S5*F*), and the biotin-labeled NEP also lacked pS6 (Fig. 5*G*).

### The M8V mutation facilitates S6 phosphorylation to downregulate NEP translocation to cell surface and extracellular vesicles

**Figure 6.**
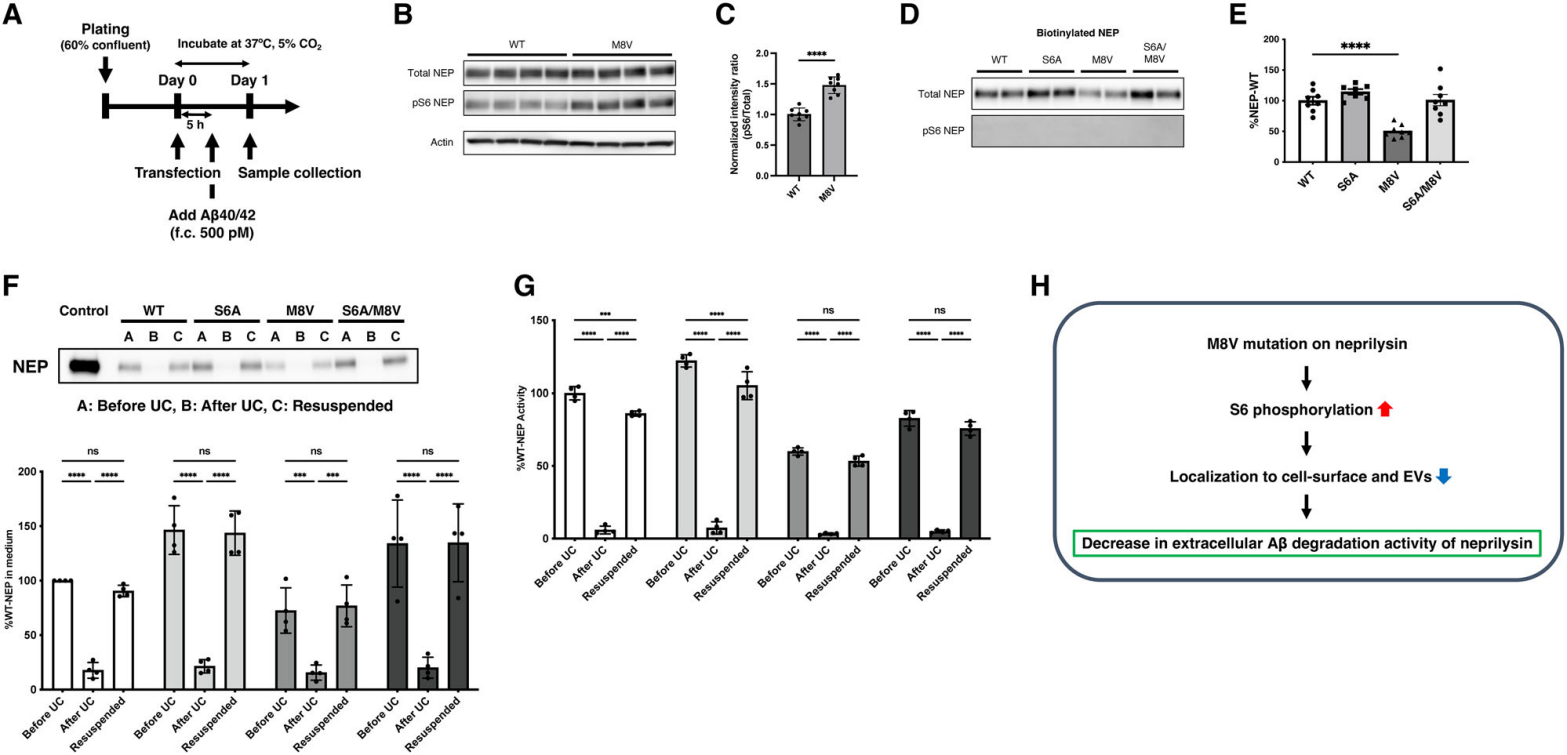
The M8V mutation upregulates NEP phosphorylation at S6 and suppresses translocation of NEP to cell surface and then to extracellular vesicles. ***A***, Experimental timeline for phosphorylated NEP analysis in SH-SY5Y cells. f.c., final concentration. ***B***, ***C***, Western blot analysis of cellular NEP phosphorylation 1 d after transfection using an antibody raised against NEP phosphorylated at S6 (Kakiya et al., 2012; ***A***) and its densitometric quantification (***B***; *n* = 8). ***D***, ***E***, Quantification of cell surface NEP protein by Western blot after biotinylation (left) and its densitometric quantification (right). ***F***, ***G***, Quantification of total NEP protein and activity in the media by Western blot (***D***) and activity assay (***E***) before ultracentrifugation (UC), after UC, and after resuspension of the centrifuged pellets (*n* = 4). ***H***, Summary of M8V effects in SH-SY5Y cells. Error bars: standard error of the mean, *p < 0.05, **p < 0.01, ***p < 0.001, ****p < 0.0001 when comparing the four groups by one-way ANOVA.

To further explore how the M8V mutation reduces extracellular Aβ degradation, we analyzed cell lysates and culture media 1 d post-transfection (Fig. 6*A*). We detected pS6 in the lysates of WT and M8V NEP-expressing cells, with a significant increase in pS6 levels in M8V-expressing cells, despite no changes in NEP expression levels or enzymatic activity (Fig. 6*B*,*C*; Fig. S5*H,I*). Immunoprecipitation using an anti-NEP antibody followed by LC-MS/MS analysis further confirmed S6 phosphorylation in both WT and M8V lysates, with a greater enrichment of phosphorylated S6 in M8V-expressing cells (Fig. S5*J*). Furthermore, NEP levels in the culture medium were reduced in M8V-transfected cells with no detectable pS6 (Fig. S5*K,L*). Cell surface biotinylation assays consistently showed reduced NEP levels on the cell surface of M8V-expressing cells, with no detectable pS6 (Fig. 6*D,E*). These findings indicate that the M8V mutation markedly enhances S6 phosphorylation, thereby impairing NEP translocation to both the cell surface and the extracellular environment. Additionally, phosphorylated S6-NEP appears to be absent from the cell surface and extracellular medium, suggesting that S6 phosphorylation restricts NEP localization to intracellular compartments.

We subsequently characterized NEP in the media, which was reported to be released from the cell surface to the extracellular space by shedding or by translocation to exosomes (Pavo et al., 2020). We confirmed no detectable alteration in the molecular weight of NEP between media and cell lysates at day 1 or 3 post-transfection, implying that NEP protein mostly did not undergo proteolytic shedding. Ultracentrifugation of the media eliminated a large portion of NEP protein and its activity therein, suggesting its translocation to extracellular vesicles (EVs). Consistently, resuspension of the centrifuged pellets resulted in full recovery of NEP expression and activity (Fig. 6*F*,*G*). There was no difference in the amount of EVs positive for CD9 and CD63, markers for exosomes (Fig. S5*M*; Kuruppu et al., 2014). These observations indicate that the M8V mutation promotes S6 phosphorylation of NEP to suppress its translocation to cell surface and EVs, resulting in decreased degradation of extracellular Aβ40 and Aβ42 (Fig. 6*H*).

### Enzymatically active NEP is present in CD9/CD63-positive extracellular vesicles

**Figure 7.**
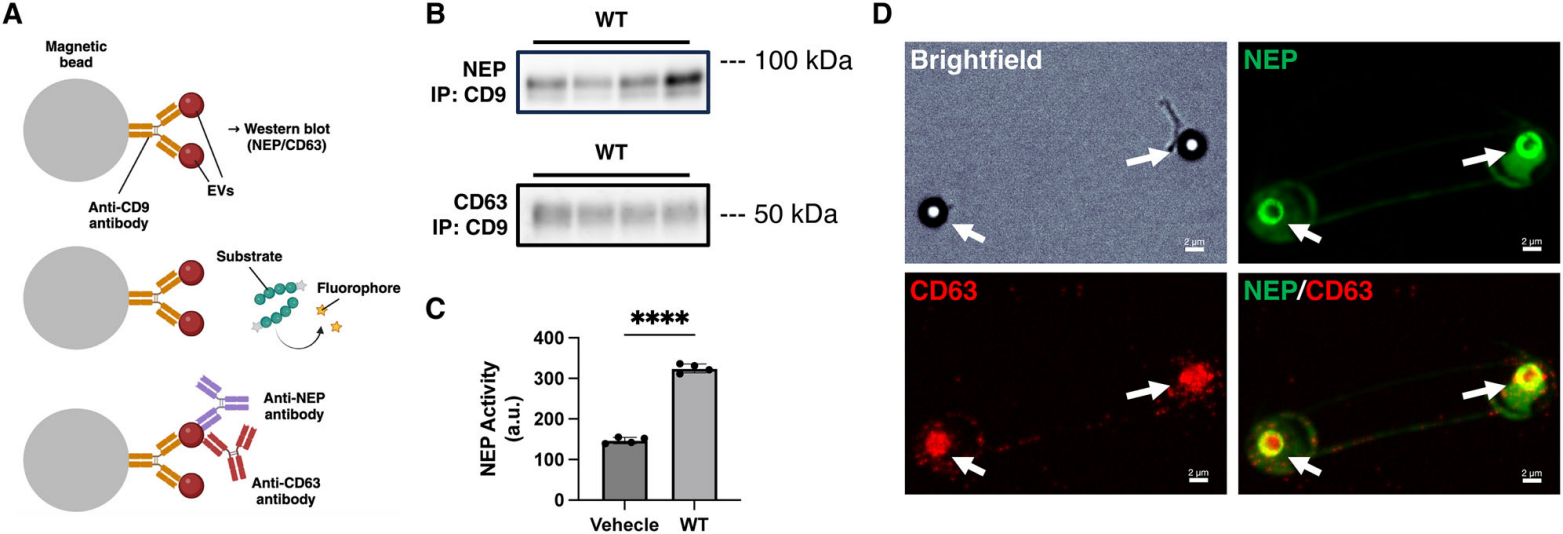
Enzymatically active NEP exists in CD9/CD63-positive extracellular vesicles. ***A***, Scheme for EVs capture analysis using CD9 antibody-coated magnetic beads. ***B***, Western blot analysis of beads-captured EVs against NEP and CD63 (*n* = 4 from independent cell cultures). IP, immunoprecipitation. ***C***, NEP activity measurement of beads-captured EVs. a.u., arbitrary unit. Error bars: standard error of the mean, ****p < 0.0001 when comparing by *t* test. ***D***, Immunofluorescence of NEP/CD63 antibodies-labeled magnetic beads.

To confirm the presence of NEP in EVs from cell culture, we isolated CD9-positive EVs using CD9 antibody-coated magnetic beads. The captured EVs were analyzed by Western blot, NEP activity assay, and immunofluorescence to assess whether they contained functional NEP and CD63 (Fig. 7*A*). Western blot analysis confirmed the presence of both NEP and CD63 in the isolated EVs, indicating successful EV isolation and NEP localization within EVs (Fig. 7*B*). Furthermore, NEP activity assays demonstrated that the NEP proteins in EVs have the activity to degrade substrate peptides (Fig. 7*C*). Immunofluorescence analysis showed colocalization of NEP and CD63 on CD9-coated beads (Fig. 7*D*). These findings confirm that enzymatically active NEP is present in CD9/CD63-positive EVs.

## Discussion

More than two decades have passed since we identified NEP as a major physiological Aβ-degrading enzyme (Iwata et al., 2000, 2001). Mathematical formulations of rate constants for the anabolism, catabolism, and clearance (to cerebral spinal fluid/plasma) of Aβ indicated that NEP activity would account for ∼50% of all Aβ clearance mechanisms given that a deficiency of NEP led to an approximately twofold increase in Aβ levels in vivo (Iwata et al., 2001; Saido, 2003b, 2024). It is notable that just a 50% increase of Aβ production results in early Aβ deposition for some cases of FAD (*APP* gene duplication; Rovelet-Lecrux et al., 2006) and Down's syndrome (Selkoe and Hardy, 2016), implying that a 50% reduction of NEP expression/activity, which increases endogenous brain Aβ levels 1.5-fold (Iwata et al., 2001), is sufficient to cause pathological Aβ deposition leading to AD development. Our present study showed that NEP is responsible for physiological and pathological Aβ metabolism as Aβ deposition was significantly accelerated in *App^NL-F^* × *Mme* KO mice. It is possible that NEP reduces GuHCl-soluble Aβ by degrading monomeric Aβ although this is difficult to prove experimentally if the GuHCl-soluble Aβ oligomers/protofibrils are sensitive to detergent-dependent dissociation.

A large body of genetic, pathological, and molecular biological evidence has established empirical roles for neuroinflammation in the pathogenesis of AD (Chen et al., 2020; Qin et al., 2021; Schwartzentruber et al., 2021; Sobue et al., 2021). Consistently, we have shown that NEP-sensitive amyloidogenic Aβ was closely associated with neuroinflammation. GFAP immunopositivity, a marker of astrogliosis, was proportionally increased with Aβ pathology in *App^NL-F^* and three other NEP- and/or IDE-deficient *App* knock-in mice. However, Iba1 immunopositivity, a marker of microgliosis, was increased in *App^NL-F^* mice but not in Aβ plaque-accumulating NEP-deficient mice. This is inconsistent with our previous report showing that *App^NL-G-F^* mice, representing a more aggressive model of Aβ amyloidosis, exhibited linearly increasing microgliosis up to 18 months of age (Masuda et al., 2016). The mechanism that accounts for this discrepancy remains elusive, but as vascular inhibition of NEP activity can influence neuropeptide levels in the heart and kidney (Bellis et al., 2020; Haynes et al., 2020; Bozkurt et al., 2023), it may indirectly influence inflammatory processes in the brain now that the interplay of CNS with peripheral organs is well recognized in the etiology of neurodegenerative disease (Saido, 2024). To further clarify the roles of NEP-sensitive Aβ deposition in neuroinflammation and behavior, it will be necessary to generate CNS-specific conditional NEP knock-out mice. It should also be pointed out that we used KO models rather than overexpression or upregulation models to investigate the physiological roles of NEP and IDE. The potential side effects of NEP upregulation in the CNS should be further studied for application of NEP activity to the treatment of preclinical AD. We observed no apparent abnormalities in the peripheral organs of NEP or IDE KO mice; however, further analysis will be needed to assess the effect of NEP deficiency on cells and organs across the entire body. Behavioral analysis revealed memory impairment in both male and female *Mme* KO mice, as well as in female *Ide* KO mice (Fig. 1*E*). While this study primarily focused on *Mme* KO mice, *Ide* KO mice may also exhibit memory deficits. However, female behavior can be influenced by hormonal cycles. In contrast, the behavioral abnormalities in *Mme* KO mice were considered reliable, as they were consistently observed in both males and females.

NEP deficiency upregulated inflammatory gene expression, while downregulating genes related to neuronal development in the cerebral cortex. Pathway analyses revealed that the *Mme* gene, along with other downregulated genes, was associated with four of the top 10 downregulated pathways, including nervous system development and neurogenesis. In contrast, the top 10 upregulated pathways were not associated with the *Mme* gene. These results suggest that NEP deficiency may lead to incomplete neuronal development, resulting in pro- or anti-inflammatory phenotypes indirectly. This may also account for the anomalous long-term memory observed in single *Mme* KO mice.

Quantification of Aβ in *Mme* KO and *Ide* KO mouse cortices by ELISA indicated that IDE only degrades TS-soluble Aβ, a result that is consistent with a previous report showing that IDE degrades Aβ monomer but not oligomer species (Walsh et al., 2002). In contrast, NEP also degrades TS-soluble Aβ, but to a lesser extent than that of IDE; however, only NEP regulates the quantity of GuHCl-soluble Aβ, implying that NEP plays a crucial role in suppressing the concentration of amyloidogenic Aβ species that cause SAD. This also implies that NEP helps suppress the formation of Aβ fibril precursors, including oligomers and protofibrils, which are considered toxic and targeted by therapeutics in AD treatment (Benilova et al., 2012; Neațu et al., 2023). Consistently, NEP deficiency resulted in a marked increase of Aβ deposition in the brains of *App^NL-F^* mice. Although *App^NL-F^* mice predominantly express Aβ42 over Aβ40 due to the Iberian mutation, which does not fully reflect physiological conditions, our findings demonstrate that NEP is involved in the degradation of both Aβ40 and Aβ42 in vivo (Fig. 3*B*). Notably, *App^NL-F^* mice appear to have human Aβ levels comparable with murine Aβ levels of WT mice (Figs. 1*B*, 3*B*).

We believe that NEP plays a more important role in the AD etiology for the following reasons. (1) Risk SNPs associated with AD have been identified in the *MME* gene but not in the *IDE* gene to our knowledge. (2) The expression of NEP in the brain declines with aging (Akiyama et al., 2001; Yasojima et al., 2001a,b; Hellstrom-Lindahl et al., 2008; Jantaratnotai et al., 2013), but that of IDE does not to our knowledge. (3) NEP is a membrane-bound enzyme expressed mainly at or near the presynapse (Fukami et al., 2002; Iwata et al., 2004), where Aβ is secreted in an activity-dependent manner (Cirrito et al., 2008; Ovsepian and O'Leary, 2016; Pignataro and Middei, 2017). In contrast, IDE is mainly a cytoplasmic enzyme. (4) NEP deficiency causes memory impairment whereas IDE deficiency does not (Fig. 1*E*). (5) Activation of dopaminergic system resulted in reduction of Aβ pathology and memory impairment in the *App* knock-in mice via increased elevation of NEP (Watamura et al., 2024).

In 12-month-old mouse brains, *App^NL-F^* × *Mme/Ide* triple mutant mice showed >8 times higher concentrations of Aβ40 and Aβ42 in TS fractions than did *App^NL-F^*, *App^NL-F^* × *Mme* KO, and *App^NL-F^* × *Ide* KO mice. This observation indicates that NEP and IDE may synergistically regulate TS-soluble Aβ species and consequently affect Aβ deposition in vivo. Both Aβ40 and Aβ42 concentrations were increased in GuHCl-soluble fractions from *App^NL-F^* × *Mme* KO and *App^NL-F^* × *Mme/Ide* KO mice, but there was no significant difference between these groups, indicating that only NEP affects the GuHCl-soluble Aβ species. The *App^NL-F^* × *Mme/Ide* KO triple mutants hold more TS-soluble Aβ40 in the brain than the other lines, in contrast with *App^NL-F^* × *Mme* KO and *App^NL-F^* × *Mme/Ide* KO double mutants that exhibit equivalent concentrations of Aβ42. This is because TS-soluble Aβ40 exists at similar levels as those of GuHCl-soluble Aβ40, whereas Aβ42 exists much more abundantly in GuHCl-soluble fractions (approximately >20-fold) than in TS-soluble fractions. This observation is consistent with the general understanding of Aβ42 as a more aggregation-prone and pathogenic species than Aβ40 and implies that NEP function becomes more important under pathological conditions, in which the Aβ42/Aβ40 ratio and quantity of GuHCl-soluble Aβ species increase (Qiu et al., 1998; Vekrellis et al., 2000; Farris et al., 2003). Based on these observations, we predict the possible presence of interactive risk alleles in the *MME* and *IDE* genes although much larger sample sizes would be necessary for such human genetic studies than the current ones (Bellenguez et al., 2022).

Recent GWAS outcomes exhibited a close association between *MME* gene variants and AD incidence (Bellenguez et al., 2022). One of the variants causes M8V amino acid substitution in NEP and has been shown to attenuate the Aβ degradation activity of NEP in cell culture medium in the present study. Cell biological analyses indicated that this M8V mutation increases S6 phosphorylation and suppresses the translocation of NEP to cell surface and EVs. This is consistent with the double S6A/M8V mutations compensating for the negative effect of M8V on NEP translocation, demonstrating an indispensable role of the phosphorylation/dephosphorylation status of NEP S6 in Aβ metabolism. We have not demonstrated the effect of the NEP M8V mutation in an in vivo paradigm, but the relevance of using the in vitro paradigm can be justified by several previous studies showing an effect of pathogenic or protective mutations in the *APP* and *PSEN1/2* genes on Aβ metabolism (Tomita et al., 1997, 1998; Wisniewski et al., 1998; Chen et al., 2012; De Strooper and Voet, 2012; Jonsson et al., 2012; Obregon et al., 2012; Bamne et al., 2014; Li et al., 2016; Xu et al., 2016; Hsu et al., 2018; Schilling et al., 2023).

Concerns have been expressed in the pharmaceutical industry about modifying NEP activity to reduce Aβ levels in the brain because NEP has been conventionally suggested to degrade various neuropeptides in vitro (Turner et al., 1996, 2000, 2001; Turner and Nalivaeva, 2006). However, Saria et al. reported that the expression levels of enkephalin, which is also an in vitro substrate peptide of NEP, remained unchanged in the cortices of NEP KO mice (Saria et al., 1997). We confirmed that NEP deficiency resulted in minimal or no alteration of somatostatin, cholecystokinin, neuropeptide Y, or substance *p* levels in the cortices and hippocampi of KO mice in our study. This is presumably because NEP degrades its substrate(s) inside secretory vesicles or on the presynaptic membrane of excitatory neurons (Fukami et al., 2002; Iwata et al., 2002, 2004), whereas neuropeptides are generally secreted from inhibitory neurons (Robbins, 1985; Epelbaum et al., 1994; Xapelli et al., 2006; Nässel, 2009), making it difficult for these neuropeptides to encounter NEP in vivo. Besides, NEP acts as a functional peptidase that degrades pathophysiologically relevant peptides in the heart and kidney: its influence in the CNS neuropeptides may be limited (Judge et al., 2015; Haynes et al., 2020). In particular, NEP present in the kidney, whose quantity is ∼100-fold larger than that of CNS counterpart (Turner et al., 1996, 2000, 2001; Turner and Nalivaeva, 2006), is likely to play more important roles. NEP inhibitors for cardiac dysfunction/failure treatment may increase the risk of developing AD if they are blood–brain barrier-permeable (Wang et al., 2019). Importantly, a series of classic enzymatic studies of NEP utilized the enzyme samples solubilized with detergent(s) that possesses easier access to TS-soluble neuropeptides in a test-tube paradigm (Turner et al., 1996, 2000, 2001). We thus propose that the membrane-bound nature of NEP be seriously considered for understanding its physical functions and pathological roles.

Rofo and colleagues successfully introduced soluble NEP into the brain parenchyma of an AD mouse model using a transferrin receptor antibody conjugate construct and showed that the soluble NEP preferentially degrades Aβ monomer (Rofo et al., 2022). However, it remains uncertain whether the introduced soluble NEP lacking the transmembrane domain degrades Aβ in a manner identical to that of endogenous NEP that preferentially proteolyzes Aβ at or near presynapse. Importantly, enkephalin is rapidly degraded by solubilized NEP in a test-tube paradigm, but not so in vivo (Saria et al., 1997). The same holds true for somatostatin, substance P, cholecystokinin, and neuropeptide Y (Fig. 2*A*).

In conclusion, we have shown here that NEP deficiency results in severe Aβ plaque deposition in *App^NL-F^* mice and that the AD risk-associated NEP mutation M8V causes a marked impairment of NEP action on Aβ metabolism. Our results strengthen the hypothesis that an aging-associated decline of NEP in the brain is likely to be one of the primary causes for SAD. Our observations also endorse the strategy to upregulate NEP expression or the activity of excitatory neurons in the CNS via somatostatin or dopamine receptor activation (Saito et al., 2005; Watamura et al., 2024). This could be considered a viable strategy to reduce Aβ deposition in preclinical AD to either halt or delay onset of the disease (Saido, 2024). Notably, administration of ʟ-DOPA to *App^NL-F^* mice for 3 months actually made some existing plaques disappear (Watamura et al., 2024), supporting the applicability of NEP-based strategies to treatment of preclinical AD.
